# Club-Foot

**Published:** 1835

**Authors:** 


### Art. IV.—Club Foot.

*Suggestions on the treatment of Club-Foot.* By a Correspondent.

From the number of uncured cases of an unfortunate class of invalids, it would seem that the attention of the medical profession should be directed to them for their relief. This is the class of persons who are born with the deformity usually denominated club foot. Whteher it has been from the neglect of their friends, in not calling for surgical aid, or whether the skill of surgeons employed has been baffled, that so many of this description of cripples are seen hobbling through our streets, I cannot say. But their having grown up uncured, I conceive is full evidence of fault somewhere. From the examinations which I have made of cases of club foot, I am of opinion, that in most instances they admitted of a complete cure, had the proper measures been taken in due season. In most cases that I have examined, there seemed to be no deficiency of parts, but a misplacement from their natural position. Whilst the bones are soft and flexible, and the ligaments susceptible of yielding to small force, this deformity is under the control of the surgeon, and at this period children born with club foot should be attended to. There are, doubtless, different successful modes of treating such cases. I have known the apparatus recommended by Dorsey used unsuccessfully. Whether this was from the fault of the apparatus or the surgeon, I cannot say; but I am inclined to believe, that there can be no shoe fitted with straps and buckles, that is so well adapted to all the circumstances of the case, as other means which we have in our power. It appears to me, that uniform pressure from the toes up the leg is desirable, and it is impossible that this should be done by buckled straps. I have been in the habit of treating the club foot in a different manner from what I have seen recommended in books, and from the success with which I have met, in this mode of treatment, I can recommend it with confidence. This mode is by the use of bandages. To illustrate it I will give the history of a case.

In 1827, A. E. was born with both feet deformed. One foot had the upper part drawn upon a line with the tibia, and the os calcis, presenting downwards, was the only part that could have been used to stand erect upon. The other foot was turned to one side, resembling a completely dislocated ancle; the side and upper part (or which should have been) occupied the place of the sole. The tibia was much curved. As soon as the mother was able to give the necessary attention, I commenced my efforts to remove the deformity. I applied two sets of easy bandages, three fourths of an inch wide, from the toes to the knees as neatly as I could. To the curved tibia a splint was then applied, and over these were applied two sets more of bandages soaked in paste made of flour. While the rollers were applying, the feet were brought to as near a natural position as moderate force would bring them, and they were retained in this position until the outer bandages became dry and firm. In two or three days the bandages were soaked in warm water and removed and again applied as before, bringing the limbs nearer to a natural position. This was done for a few weeks till the limbs were entirely cured of deformity. The latter part of the time did not require frequent changes of the dressings. The paste bandages applied in this manner afforded a strong and unyielding covering, keeping the limbs in the exact posture desired, incapable of yielding to any force which might happen from accident; setting easy and allowing the most free circulation, producing no irritation, and giving sufficient space for the growth of the limb, from the elasticity of the dry strips between the limb and the external covering. At the present time, it would be impossible for the most careful observer to detect the least deformity, either in the feet or the legs.

